# From genes to functional classes in the study of biological systems

**DOI:** 10.1186/1471-2105-8-114

**Published:** 2007-04-03

**Authors:** Fátima Al-Shahrour, Leonardo Arbiza, Hernán Dopazo, Jaime Huerta-Cepas, Pablo Mínguez, David Montaner, Joaquín Dopazo

**Affiliations:** 1 Bioinformatics Department, Autopista del Saler 16, E46013, Valencia, Spain; 2 Functional Genomics Node, INB-CIPF Centro de Investigación Príncipe Felipe (CIPF), Autopista del Saler 16, E46013, Valencia, Spain

## Abstract

**Background:**

With the popularisation of high-throughput techniques, the need for procedures that help in the biological interpretation of results has increased enormously. Recently, new procedures inspired in systems biology criteria have started to be developed.

**Results:**

Here we present FatiScan, a web-based program which implements a threshold-independent test for the functional interpretation of large-scale experiments that does not depend on the pre-selection of genes based on the multiple application of independent tests to each gene. The test implemented aims to directly test the behaviour of blocks of functionally related genes, instead of focusing on single genes. In addition, the test does not depend on the type of the data used for obtaining significance values, and consequently different types of biologically informative terms (gene ontology, pathways, functional motifs, transcription factor binding sites or regulatory sites from CisRed) can be applied to different classes of genome-scale studies. We exemplify its application in microarray gene expression, evolution and interactomics.

**Conclusion:**

Methods for gene set enrichment which, in addition, are independent from the original data and experimental design constitute a promising alternative for the functional profiling of genome-scale experiments. A web server that performs the test described and other similar ones can be found at: .

## Background

Genes do not operate alone within the cell, but in a intricate network of interactions that we have only recently started to envisage [1-3]. It is a widely accepted fact that coexpressing genes tend to be fulfilling common roles in the cell [4,5]. Moreover, coexpression seems to occur, in many cases, in contiguous chromosomal regions [6] and furthermore, recent evidences suggest that functionally related genes map close in the genome, even in higher eukaryotes [7]. Many higher-order levels of interaction are continuously being discovered and even complex traits, including diseases, have started to be considered from a systems biology perspective [8]. In this scenario, a clear need exists for methods and tools which can help to understand large-scale experiments (microarrays, proteomics, etc.) and to formulate genome-scale hypothesis (evolution, architecture of the interactome, etc.) from a systems biology perspective [9]. Thus, the functional interpretation of genome-scale data in this context must be taken within a systems biology framework, in which the collective properties of groups of functionally-related genes are considered.

DNA microarray technology can be considered a paradigm among genome-scale experimental methodologies. Its extensive use has fuelled the development of tools for the functional interpretation of such experiments. These tools study the enrichment of functional terms shown by groups of genes defined by experimentally determined gene expression levels. Programs such as ontoexpress [10], FatiGO [11], GOMiner [12], etc., can be considered representatives of a family of methods designed for this purpose [13,14]. The difficulties for defining repeatable lists of genes of interest across laboratories and platforms using common experimental and statistical methods [15] has led several researchers to propose different approaches which aim to select blocks of genes with known common functional properties.

Thus, the Gene Set Enrichment Analysis (GSEA) [16,17], although not free of criticisms [18], pioneered a family of methods conceived to search for groups of functionally related genes with a coordinate over- or under-expression across a list of genes, ranked by their differential expression, coming from microarray experiments. Different tests have recently been proposed for this purpose [19-24] and also for ESTs [25]. Nevertheless, it is surprising that, despite the abundance and availability of genome-scale data, the notion of testing entities more complex than single genes (such as blocks of functionally related genes) has not been applied in fields other than microarray data analysis. In fact, any genome-scale data in which some measurement is available for individual genes can be analysed in a similarly conceptual way.

Here we "officially" present the FatiScan program, which implements a segmentation test [19] that allows studying many relevant functional terms, which include Gene Ontology (GO) [26], KEGG pathways [27] and many others, along with a sophisticated system for the visualisation of results. Although FatiScan had been mentioned in previous papers dealing with generalities of the GEPAS [28] and Babelomics [29,30] program packages, a proper detailed description of FatiScan and their possibilities was not available to date. FatiScan can deal with ordered lists of genes independently from the nature of the experiment that originated the data or the method used to rank the genes. This interesting property allows for its application to other type of data apart from microarrays. We show how FatiScan can be applied to different genome-scale datasets such as protein-protein interaction networks or to test functional evolutionary hypotheses. We also show how conclusions on the molecular roles fulfilled by the genes can be reached by taking into account the functional interplay of genes in the cell as defined by their shared biological properties.

### Threshold-based functional profiling

The interpretation of genome-scale data is usually performed in two steps: in a first step, genes of interest are selected (for example, in microarray experiments, because they are significantly over- or under-expressed when two classes of experiments are compared), and then, the enrichment of any type of biologically relevant term in these genes with respect to a background (typically the rest of the genes) is studied. In the active field of microarray data analysis, there are different available tools, such as Oncomine [10], FatiGO [11] and others [13,14], that use different functionally relevant terms taken from different curated repositories (GO [26], KEGG pathways [27], etc.) It has been noted that this strategy causes an enormous loss of information due to the large number of false negatives that are accepted in order to preserve a low ratio of false positives (and the noisier the data the worse the effect) [16,19,30].

### Threshold-free functional profiling

**Figure 1:**
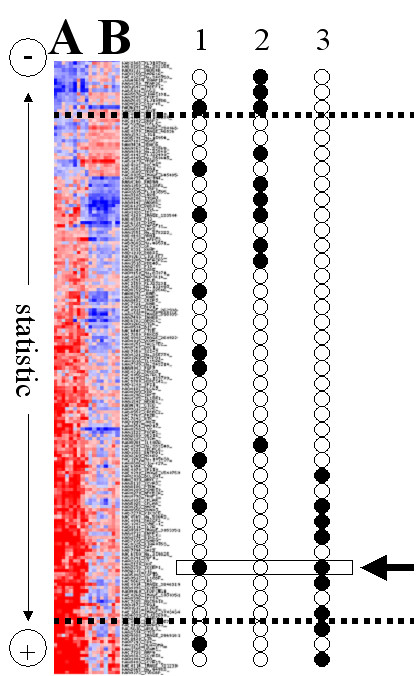
Threshold-free functional analysis. A list of genes is ranked by their differential expression between two experimental conditions (**A **and **B**) using, for example, a t-test which is applied individually to each gene. Columns **1**, **2 **and **3 **represent the position of the genes belonging to three different functional classes (e.g. GO terms, etc.) across the arrangement. The first functional class is completely uncorrelated with the arrangement, while functional classes **2 **and **3 **are clearly associated to high expression in the experimental conditions **B **and **A**, respectively. Dotted lines represent a threshold based on the individual t-tests with some adjustment for multiple testing. The arrow makes reference to the multi-functional character of the genes: a gene can belong to more than one functional class. In this case the gene pointed out by the arrow is in this position not because of its membership to functional class **1 **but because is fulfilling the role corresponding to functional class **3**, which is related to high expression in experimental condition **A**.

Under a systems biology perspective, a threshold-based approach to understanding the molecular basis of a genome-scale experiment is far from being efficient. Methods that draw inspiration from systems biology focus on functional classes such as blocks of genes that act cooperatively rather than on single entities such as genes. These strategies use lists of genes ranked by any biological criteria (e.g. differential expression when comparing cases and healthy controls, genes with different evolutionary rates, etc.) and directly search for the distribution of blocks of functionally related genes across such list [16,19-24]. Any macroscopic observation that causes this ranking in the list of genes will be a consequence of the cooperative action of genes arranged into functional classes (GO, pathways, etc.) Each functional class "responsible" for the macroscopic observation will, consequently, be found in the extremes of the ranking with the highest probability. Figure 1 illustrates this concept. Let's imagine that a list of genes is ranked by differential expression between two experimental conditions (A and B in the figure). If the position of the genes belonging to different functional classes is studied (columns 1, 2 and 3 in Figure 1) it is evident that the functional class represented in the first column is completely uncorrelated with the arrangement, while the other two are clearly associated to high expression in the experimental conditions B and A, respectively. If, for example, the two experimental conditions were diseased versus healthy controls, column 1 could correspond to a functional class related to housekeeping processes. Consequently, the genes corresponding to this functional class would be active in both conditions (healthy and diseased) and will be scattered across the list. Conversely, columns 2 and 3 would correspond to biological processes much more active in diseased cases (B) or in healthy controls (A), respectively. If thresholds were imposed to select genes differentially expressed (dotted lines in Figure 1), and genes over this threshold were compared to the rest for enrichment in these functional classes, the chance of finding a significant enrichment in this pre-selection of genes would be much lower, if not null. The imposition of a previous threshold based on experimental values that ignores the cooperation among genes is thus avoided under this threshold-free perspective.

## Implementation

### FatiScan: the segmentation test implemented

The aim of the test is to find functional classes, namely blocks of genes that share some functional property, showing a significant asymmetric distribution towards the extremes of a list of ranked genes. This is achieved by means of a segmentation test, which consists on the sequential application of a Fisher's exact test over the contingency tables formed with the two sides of different partitions made on an ordered list of genes. The Fisher's exact test finds significantly over or under represented functional classes when comparing the upper side to the lower side of the list, as defined by any partition. Previous results show that a number between 20 and 50 partitions often gives optimal results in terms of sensitivity and results recovered [19]. Given that multiple functional classes (*C*) are tested in multiple partitions (*P*), the unadjusted p-values for a total of *C *× *P *tests are corrected by the widely accepted FDR [31]. Performing *C *× *P *tests would correspond to the worst scenario, in a situation in which no *a priori *biological knowledge of the system is available. Usually many functional classes can initially be discarded from the analysis due to prior information or just by common sense.

A fundamental advantage of the FatiScan methods is that it does not depend on the original data from which the ranking of the list was derived. The significance of the test depends only on the numerical values used to rank the genes in the list and the strategy used for performing the partitions. This means that, in addition to DNA microarray data, this method can be applied to any type of genome-scale data provided that an experimental or theoretical value can be obtained for each gene, and genes can be ranked according to such value.

### FatiScan: the program

The FatiScan program [32] is a web-based application that can be found within the Babelomics environment [33] for functional analysis of large-scale data, which is in turn, integrated in the GEPAS environment [28,34-36] that provides a whole set of tools and data bases for microarray data analysis.

#### The input form and the data

**Figure 2:**
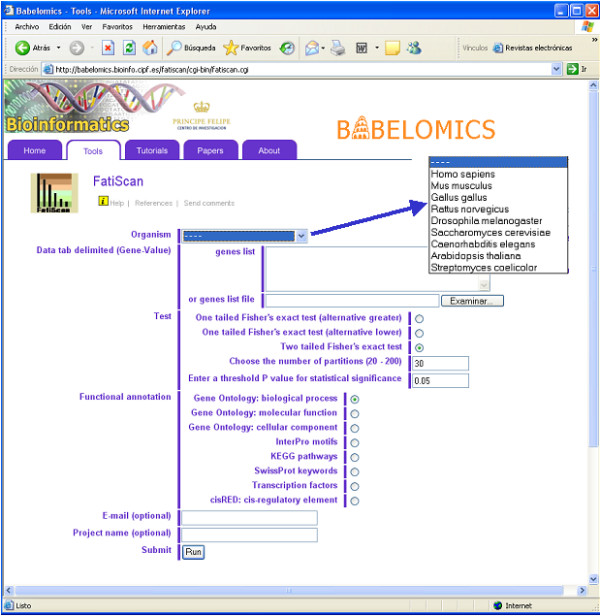
Interface to the FatiScan program displaying the available model organisms.

Figure 2 shows the application's main page. This interface allows entering the data by just pasting it into the box or uploading it from a text file. The data format is straightforward: There are two columns, the first one corresponds to the gene identifiers, and the second one to the value of the parameter used to rank the list. The example shown below corresponds to the case study presented (see section "Functional analysis of microarray experiments"), in which the ordered list has been generated by means of a t-test for differential gene expression between healthy controls (class NTG in the list stands for normal tolerance to glucose) and diabetics, as well as diabetic associated diseased cases (DM2/IGT stands for Diabetes Mellitus 2 and Impaired glucose tolerance patients) taken from literature [16]. Most rows starting by the # symbol are used to add comments to the dataset and are ignored by the program. The exception are rows starting with #TOP and #BOTTOM tags. These two tags may be used to describe the biological meaning attached to the order of the genes and are used by FatiScan to make the result plots more understandable to each particular user.

#TOP genes more expressed in 'NGT' class

#BOTTOM genes more expressed in 'DM2/IGT' class

219666_at 4.5308394432

200941_at 4.2713012695

221559_s_at 3.957174778

200885_at 3.9373190403

219244_s_at 3.7689547539

213348_at 3.6691555977

220547_s_at 3.6681389809

: :

217741_s_at -3.929725647

201539_s_at -3.9333205223

216651_s_at -3.9856903553

213710_s_at -4.8559875488

214587_at -5.0099239349

The organism to which the gene identifiers refer to must be selected from a list of choices that includes the most representative species and model organisms (see below).

At the bottom of the form the user can choose to provide an email address, to which the results will be automatically submitted, so that it is not necessary to wait online until the completion of the test (which sometimes may take a few minutes). A name for the project is optional, but quite useful when many tests are simultaneously being performed.

#### The tests and the statistic

**Figure 3:**
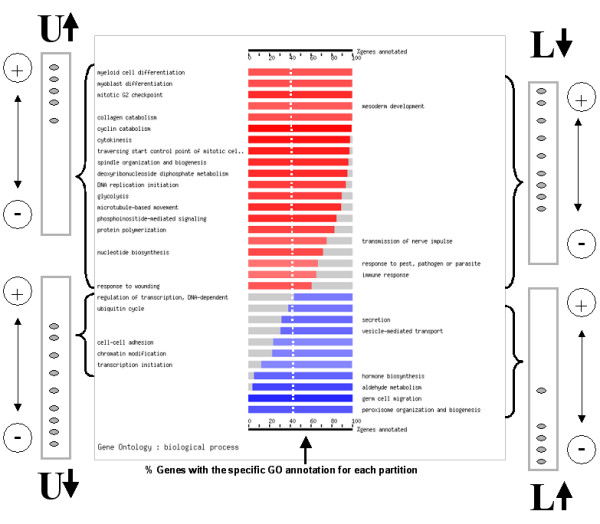
A general picture of the results of the FatiScan program. Over- and under-representations of functional classes in both tails of the list of arranged genes can be detected. **U-arrow up**: functional classes over-represented in the upper part of the list. **U-arrow down**: functional classes under-represented in the upper part of the list. **L-arrow up**: functional classes over-represented in the lower part of the list. **L-arrow down**: functional classes under-represented in the lower part of the list. See text for the different choices of tests that detect the different cases.

Figure 2 also displays the available options for the test:

• **One tailed Fisher's exact test (alternative greater)**: will detect functional classes over-represented in the upper part of the list or under-represented in the lower part. (corresponding to U-arrow up and L-arrow down labels in Figure 3).

• **One tailed Fisher's exact test (alternative lower)**: will detect functional classes over-represented in the lower part of the list or under-represented in the upper part. (corresponding to U-arrow down and L-arrow up labels in Figure 3).

• **Two tailed Fisher's exact test**: will simultaneously detect all the four scenarios of the two previous options. (all the cases in Figure 3).

Depending on the nature of the experiment and the arrangement, one of the three options can have more biological meaning than the other two.

As previously described, the FatiScan method proceeds through a series of partitions. The user has the option of using different number of partitions, nevertheless, previous studies show that 30 is a reasonable choice and so it is set as a default value [19].

Finally a p-value defining the threshold of statistical significance for the test can also be entered here.

#### The functional data

Different repositories of functional and biological information are used to define the functional classes used in the test. We have collected information from different repositories for several model organisms (*Homo sapiens, Mus musculus, Rattus norvegicus, Drosophila melanogaster, Caenorhabditis elegans, Saccharomyces cerevisiae *and *Arabidopsis thaliana*), which have been cross-referenced using Ensembl [37] identifiers. The repositories used are:

• GO which is probably the most successful among the initiatives for the standardisation of the nomenclature of biological processes, molecular functions and subcellular location (its three main ontologies) [26].

• InterPro [38] which is a database of protein families, domains and functional sites from which identifiable features (motifs) found in known proteins can be used to predict the possible functionality of unknown protein sequences

• The SwissProt [39] database, now part of the Unitprot resource, which contains a field called keywords for each entry that implements a controlled vocabulary of words, many of them (although not all) with functional meaning.

• KEGG pathways [27] which is a collection of pathway maps representing the knowledge on the molecular interaction and reaction networks for Metabolism, Genetic Information Processing, Environmental Information Processing, Cellular Processes and Human Diseases.

• Transcription factor binding sites predicted using Transfac^® ^[40]. Transcription factors (TFs) are assigned to genes if their corresponding predicted transcription factor binding sites (TFBS) are found in the 10 kb 5' region of the genes. The search is carried out by the Match program [41], using only high quality matrices and with a cut-off to minimize false positives, from the Transfac database. TFBSs are only available for human and mouse.

• CisRed [42] which is a database for conserved regulatory elements predicted in promoter regions using multiple discovery methods. In theory, all the Transfac^® ^predictions should be a subset of these regulatory elements, but in practice the overlap is not complete. For this reason the Transfac^® ^predictions are independently provided. CisRed tables are only available for humans.

The tables of correspondence from genes to functional terms derived from these repositories are used to define different categories of functional classes for further use in the functional analysis of the experiments. These functional classes are composed by genes that share the same functional term, depending therefore, on the category (repository) used for the annotation, genes will be part of different functional classes. Given their multi-functional character (e.g. a number of genes simultaneously belong to the functional classes "transcription factor" and "DNA binding") genes can indeed belong to more than one functional class.

#### The results

**Figure 4:**
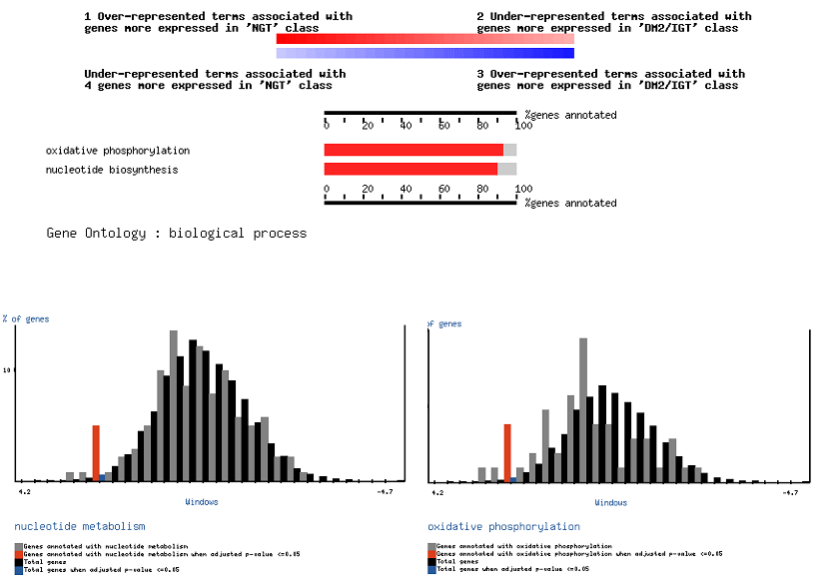
Graphical results of FatiScan. Upper part: a summarised view of all the functional classes found. In the case of GO, only the deepest significant terms in the hierarchy are displayed. Lower part, comparison of the distribution of the two GO terms found significantly over-represented in the upper part of the list with respect to the background of GO terms in the rest of genes.

Once the organism, the category and the test have been selected, the program can be ran. The results will report the functional classes, corresponding to the category selected, which have been found to be asymmetrically distributed towards the extremes of the list. Figure 4 shows an example (more extensively discussed below, in the section "Differential gene expression in human diabetes samples") in which the two general processes found (*oxidative phosphorylation *and *nucleotide biosynthesis*, belonging to the biological process ontology of GO) are shown (upper part of the figure). In addition, the user can visualise the distributions of both GO terms with respect to the background distribution in the rest of genes. Also, information on the genes belonging to the different functional classes (GO terms in this case) is listed.

### Testing terms across the GO directed acyclic graph

**Figure 5:**
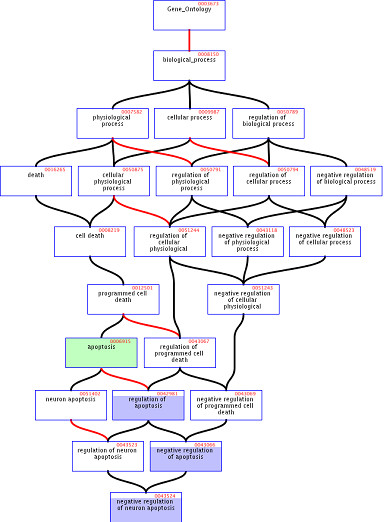
A small segment of the DAG GO hierarchy connecting the top of the *biological process *category to the term *negative regulation of neuron apoptosis*.

Most of the functional or biological terms used for the definition of functional classes are "flat", that is, there is no structure behind them. For example, KEGG pathways are independent from each other (at least, information on dependencies is not included in the database). This is not the case of GO, in which the biological knowledge is represented as a tree (more precisely as a directed acyclic graph, DAG, in which a node can have more that one parent). In the GO hierarchy (see Figure 5), upper nodes represent more general concepts and, as the DAG is traversed towards deeper levels, the definitions are more and more detailed (e.g. GO terms in the tree from more general to more detailed would be: *biological process *> *physiological process *> *death *> *cell death *> *programmed cell death *> *apoptosis*, etc.) Since genes are annotated at different levels it is common to use the inclusive analysis [43] (or custom level of abstraction [13]) instead of using directly the level of annotation of the genes. In this case we consider that a gene annotated to a given level, is automatically annotated to all the upper levels in the hierarchy (e.g., a gene annotated as *apoptosis *is, obviously, a gene of *programmed cell death*, of *cell death*, and so on upwards). Then, if *cell death*, is chosen as the level of abstraction to be analysed, genes annotated as *programmed cell death*, *apoptosis*, and so on downwards, will be annotated as *cell death*. This abstraction can also be found in the GoSlim project [44] of the Gene Ontology project. Using this strategy increments the efficiency of the test given that there are less terms to test and more genes per term, but the selection of the level is arbitrary. Here we have implemented the Nested Inclusive Analysis (NIA), in which the test is recursively conducted at different abstraction levels until the deepest level in which a statistically significant enrichment is reached. Only this deepest level is reported in the summary (which would correspond to the upper part of Figure 4). The results obtained for the upper levels can also be found in the detailed report of the results. In this way, both the aspects of efficiency and that of reporting the GO terms with the highest descriptive precision are optimised. Figure 5 shows an example in which the GO terms *apoptosis*, *regulation of apoptosis*, *negative regulation of apoptosis *and *negative regulation of neuron apoptosis *were significant. In this case only *negative regulation of neuron apoptosis *will be reported in the summary of functional classes (upper part of Figure 4).

## Results and discussion

### Functional analysis of microarray experiments

#### Functionally related genes show coordinate expression

It is a long recognized fact that genes with similar overall expression often share similar functions [4,45-47]. This observation is consistent with the hypothesis of modularly-behaving gene programmes, where sets of genes are activated in a coordinated way to carry out functions. Therefore, if a list of genes ranked according to their magnitude of differential expression between two experimental conditions is studied, the genes fulfilling molecular roles that account for the experimental differences will most probably be found at the extremes of the list (that is, these blocks of functionally related genes will be co-ordinately over- or under-expressed as a whole system) [16,19].

#### Differential gene expression in human diabetes samples

We have used a study of gene expression in a case-control study in human diabetes [16] where two categories of experiments (17 controls with normal tolerance to glucose versus 26 cases composed by 8 with impaired tolerance and 18 with type 2 diabetes mellitus, DM2) were defined. A comparison between both categories did not detect even a single differentially expressed gene.

We ordered the genes according to their differential expression between cases and controls. A t-test, as implemented in the T-Rex tool from the GEPAS package [28,34-36] was used for this purpose. The value of the statistic, which accounts for the differential expression of individual genes among cases and controls, was used as the ranking criteria for ordering the list. As in the original analysis [16] we were unable of finding individual genes with a significant differential expression (differentially expressed genes with an adjusted p-value lower than 0.05).

Nevertheless, our aim was not to test individual genes but functional classes. To this end, a total of 50 partitions of the ranked list were analysed with the FatiScan algorithm for over- or under-expression of KEGG pathways and GO terms. The following KEGG pathways were found to be significantly over-expressed in healthy controls versus diseased cases: *oxidative phosphorylation*, *ATP synthesis *and *Ribosome*. Contrarily, *Insulin signalling pathway *was significantly up-regulated in diseased cases. When GO terms were analysed we have found as significantly up-regulated in healthy controls:*oxidative phosphorylation *(GO:0006119), *nucleotide biosynthesis *(GO:0009165) (biological process ontology), *NADH dehydrogenase (ubiquinone) acivity *(GO:0008137), *nuclease activity *(GO:0004518) (molecular function ontology) and *mitochondrion *(GO:0005739) (cellular component ontology). Some of the terms were redundant with the KEGG pathways, although here we have also found the *ubiquinone *class, which does not appear in KEGG. Since FatiScan implements more functional terms we have also analysed Swissprot keywords and we have found *Ubiquinone*, *Ribosomal protein*, *Ribonucleoprotein*, *Mitochondrion *and *Transit peptide *as over-expressed in healthy controls versus disease cases.

As an example, Figure 4 (bottom) shows the distribution of the GO terms *oxidative phosphorylation *and *nucleotide biosynthesis *compared to the background distribution of GO terms. A clear trend to the over-expression of the complete pathway in high values of the t statistic, corresponding to genes over-expressed in healthy controls, can be clearly observed.

Other alternative methods raised similar results. *Oxidative phosphorylation *and *mitochondrion *is found by GSEA [16], PAGE [24] and other statistics [22]. *Nucleotide biosynthesis *can be assimilated to other functional classes, based on a set of functional categories developed by [17] and found by these three methods [16,22,24]. The rest of functional classes were only found by FatiScan.

#### Beyond the categorical classes: study of the relationship between functional blocks of genes and survival, a continuous variable

There are experimental designs which do not involve discrete variables (e.g. categories such as cases and controls) but where instead, each experiment is associated to a continuous variable such as the level of a metabolite, time, etc. Survival is a special case of continuous variable of high relevance in clinics that can easily be studied with FatiScan. Other methods based on models need to specifically model each type of variable differently. For example Goeman et al., proposed two different models, one for discrete [21] variables and another one for survival [20]. We have used a series of microarray experiments with detailed survival information on patients of hypopharyngeal cancer [48] to show how Fatiscan can easily be used in the context of survival analysis.

The dataset used (GEO [49] record GDS1070) contains 34 hybridisations from tumours. The samples were taken from patients before undergoing surgery for hypopharyngeal cancer. Information about the overall survival time of patients as well as their follow up status after surgery is available [48].

Our aim was to determine the functional basis of survival at molecular level. This involves finding the functional classes with an overall expression related to the survival times of patients. Consequently, we had to create a list of genes arranged by this criterion.

To achieve this we used a Cox Proportional-Hazards model to study how the expression of each gene across patients is related to their survival times. This methodology models the logarithm of the population hazard function as a linear function of gene expression. It can handle censored data, i.e. samples for which the exact survival time is unknown, but for which it is only known that the patient is still alive at a certain time. [50]. As the hazard function assesses the instantaneous risk of dying, positive slope coefficients in the linear part of the model correspond to genes for which an increase in expression is related to shorter survival times. Reciprocally, genes with negative slope coefficients are those in which expression decreases as the survival time of the patient increases. We can use these linear coefficients (divided by their standard error i.e. we use the Wald statistic to test for the null hypothesis that the coefficient is zero) to rank our genes from those in which an increased expression is more associated with early death (higher positive statistic) to those in which an increased expression is associated to longer survival times (lower negative statistic)

Once the list of genes ordered according their relationship with survival time was obtained, the Fatiscan program was used to search for functional classes significantly associated to the genes on the top of the ranking. Such functional classes will be those with overall up-regulated expression in the patients with lower survival values. In the same way, the functional classes associated to the bottom of the ranking will be those with down-regulated expression in the patients with lower survival (or up-regulated in the patients with a larger life expectancy).

In this example, the following GO terms corresponding to the *biological process *ontology were found as significantly associated to low survival: *M phase of mitotic cell cycle *(GO:0000087) and *regulation of cell cycle *(GO:0051726), clearly related to proliferation; *cellular localization *(GO:0051641), defined as transport and/or location related to construction of new cellular structures, which is obviously related to proliferation as well; and *macromolecule metabolism *(GO:0043170) and *primary metabolism *(GO:0044238), which also correspond to the generation of new cellular components. As in many cancers we have also found *antigen processing *(GO:0030333) and *antigen presentation *(GO:0019882), which are not related to cancer cells themselves but to the surrounding cells sampled in the biopsies. Actually, almost a 30% of the cells sampled in the biopsies in this work were normal cells [48], which is a quite common scenario in many similar studies. Conversely, we have found terms related to long life expectancy, associated to the normal functioning of tissues where the cancer arises, as well as the formation of complex structures (such as nervous system or epidermis) within it. These are: *nervous system development *(GO:0007399) *muscle development *(GO:0007517) *regulation of organismal physiological process *(GO:0051239), *muscle contraction *(GO:0006936), *epidermis development *(GO:0008544). The example clearly shows how this method detects functional blocks of genes activated in the opposite satiations, long and short life expectancy, when genes are arranged based on these criteria.

### Functional analysis of selective pressures in the human genome

#### Positive selection and non-synonymous rate acceleration in human

Evolution has been carrying out knock-out experiments for more that 3,500 million years and the results can be read in the DNA, its laboratory notebook. Therefore, the signs of selection can give a great deal of information on the functional molecular modules that have shaped present day organisms. Threshold-free tests for functional classes can also be successfully applied to the formulation and contrast of genome-scale hypothesis in the evolutionary context. As an example we will study one of the major challenges in evolutionary biology: what are the functional basis of humanness at the molecular level and how evolution has shaped them. Recent efforts at a genomic scale have been conducted to elucidate the intricacies of human evolution by means of comparing rate differences and positive selection in human genes against their homologues in other fully sequenced species [51-54]. Nevertheless, beyond some conjectures, few significant conclusions about the functional roles fulfilled by the genes under different types of selective pressures could be derived from these studies. One main reason that account for the failure in finding a functional interpretation to the human evolution comes, most probably, from the fact that these studies followed an inefficient threshold-based, two-steps approach.

The hypothesis we aim to test in this study is not about individual genes, but about functional classes. Mutations occur on single genes but natural selection acts on phenotypes by operating on whole sub-cellular systems. Mutations in genes either remain finally fixed or disappear because of their beneficial or disadvantageous effect, respectively. This effect on the function of individual proteins can only be understood in the context of the system (e.g. a pathway, GO functional roles, etc.) in which the proteins are involved. If a list of genes arranged by some parameter that accounts for their evolutionary rates is examined, it is expectable that genes belonging to pathways or functional classes favoured or disfavoured by selection will tend to appear towards the extremes.

#### Ranking genes by selective pressures

A powerful approach to detect molecular evolution by positive selection is based on the comparison of the relative rates of synonymous (**Ks**) and non-synonymous (**Ka**) substitutions [55]. The ratio of these values, the (**ω **= **Ka**/**Ks**) is a widely accepted measure of the selective pressure. If non-synonymous mutations are deleterious, purifying selection will reduce their fixation rate and **ω **will be lower than one, whereas if non-synonymous mutations are advantageous, they will be fixed at a higher rate than synonymous mutations, and **ω **will be greater than one. Contrarily, an **ω **ratio equal to one is consistent with neutral evolution. A whole-genome analysis of selection allows ranking the genes according the value of their **ω **parameter.

Maximum likelihood estimations of **Ka **and **Ks **were computed for each ortholog in the human lineage under a free branch model using CodeML in the PAML program [56].

Ortholog annotations for the subset of 20,469 "known" Ensembl human protein-coding genes from the Ensembl v.30.35h *H. sapiens *database [57] were retrieved from the Ensembl-Compara database v.30 [37]. Coding sequences for the proteins represented by the largest transcript of each ortholog were retrieved from the Ensembl databases (Human: v.30.35c, Chimp: v.30.2, Mouse: v.30.33f, Rat: v.30.34, Dog: v.30.1b). DNA coding sequences were aligned using ClustalW [58] using the translated protein sequences as templates. Codons containing gaps were removed. Alignments smaller than 50 bp were excluded from the analysis. Thus, a total of 11,102 genes where used for the computation of the normalized non-synonymous rate of evolution, **ω**, in the human lineage. From this list, genes with **ω **< 0.0001 were excluded, leaving a final set of 5,648 genes. The value of **ω **was used to rank the list of genes.

#### Systems biology meets evolution: selective pressure over sets of functionally related genes

**Figure 6:**
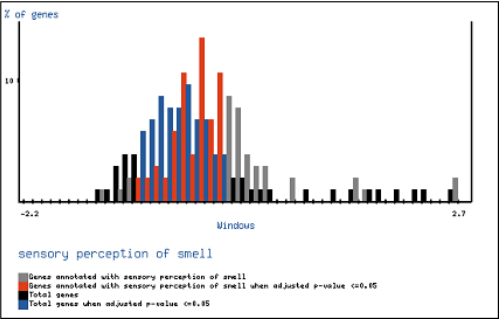
FatiScan analysis of the comparison between the background distribution of GO terms (black and blue bars) and the distribution of *sensory perception of smell *GO term (grey and red bars). The last distribution is clearly shifted towards highest values of **ω **(horizontal axis). The colours black/blue and grey/red make reference to the **ω **values for which the partitions were found to be significant.

The ordered list was log-transformed to produce a linear scale, and 50 partitions were taken for the analysis. When the FatiScan test is applied we have found the following GO terms significantly cumulated at the extreme of the distribution corresponding to the highest **ω **values: *sensory perception of smell *(GO:0007608), *sensory perception of chemical stimulus *(GO:0007606) and *G-protein coupled receptor protein signalling pathway *(GO:0007186). The FDR-adjusted p-values corresponding to the most significant partitions were 1.3 × 10^-5^, 0.0014 and 0.0095, respectively. Figure 6 shows the distribution of the genes belonging to the GO class *sensory perception of smell *(GO:0007608) across the range of genes. Clearly the GO class is shifted towards high **ω **values.

Different authors [51,52] using a threshold-based, two-steps approach, claimed to have found these GO categories significantly over-represented among positively selected genes in humans. Nevertheless, if the associated p-values were properly adjusted for multiple testing, the GO categories previously found become non-significant. We show here how the application of the FatiScan threshold-free test circumvents the problems of significance found by other authors and report as significant the classes suggested (but not statistically supported) by previous analyses.

### Functional analysis of a network of protein-protein interactions

#### Correlation between functional properties and connectivity (as measured by degree) in the human interactome

Recently, two genome-scale analysis of protein interactions have provided for the first time an extensive vision of the human interactome [1,2]. Protein interactions are on the basis of many cooperative functional processes and, consequently, some properties of networks are expected to be correlated with particular functions [59].

The first obvious property to be studied in a network is the connectivity. Many functions must occur through the formation of protein complexes [60] or via signalling pathways. Therefore, a correlation between connectivity and some molecular processes can be expected for a significant number of cases. Here we have used the interactions among human proteins stored in the DIP database [61] plus interactions predicted from sequence/structure distant patterns [62], which sum up to a total of 75,437 interactions in which 3,430 proteins are involved. The degree of interaction was estimated for each protein using the program PIANA [63,64] simply by counting the number of edge-ends in the interactome at each node. Proteins were then ranked according to the number of interactions displayed in the inferred interactome.

#### Functional terms significantly associated to connectivity in the human interactome

The analysis of the distribution of GO terms across the list of genes ordered by their connectivity (degree) provides an idea of which biological processes operate through protein complexes or transient aggregations of proteins and which ones operate through a few interactions such as some steps of signalling cascades or similar. The list ordered by the number of observed and predicted interactions was divided into ranks corresponding to: more than 500 interactions, between 500 and 400, and so on until 100. Then, the list was divided in chunks of ten interactions (between 100 and 90, between 90 and 80, etc). After the application of the FatiScan method to the 14 partitions so defined, the following terms significantly associated to a high number of interactions were found: *Disassembly of cell structures during apoptosis *(GO:0006921), *cAMP biosynthesis *(GO:0006171), *cGMP biosynthesis *(GO:0006182), *regulation of embryonic development *(GO:0045995), *fibrinolysis *(GO:0042730), *cytolysis *(GO:0019835), *complement activation, classical pathway *(GO:0006958), *digestion *(GO:0007586), *proteolysis *(GO:0006508), *neuropeptide signalling pathway *(GO:0007218). Many of them clearly operate though complexes or, alternatively, involve aggregations of a large number of proteins (even for disaggregation, like in the case of the first term). The case of *neuropeptide signalling pathway *is defined as: "The series of molecular signals generated as a consequence of a peptide neurotransmitter binding to a cell surface receptor" making reference to connectivity at the receptor level.

On the other hand, there were GO terms significantly associated to low number of interactions: *cell-cell signaling *(GO:0007267), *cell cycle *(GO:0007049), *protein amino acid phosphorylation *(GO:0001932), *transcription from RNA polymerase II promoter *(GO:0006366), *DNA repair *(GO:0006281), *chemotaxis *(GO:0006935) and *positive regulation of cell proliferation *(GO:0008284). General processes of cell signalling appeared here. Despite that these processes can involve many proteins, their interaction occurs through a sequence of steps. For example, cascades of successive contacts in contrast to the case of protein complexes where each protein binds to many others simultaneously. Phosphorylation, transcription, and DNA repair would fall in a similar class of poorly connected processes.

We have shown how a simple property of a network of interactions can be associated to functional processes through a systems biology inspired procedure such as FatiScan.

### An approximation to the relative performances of different Threshold-free methods

In order to check whether the findings of FatiScan were similar or not to other alternative threshold-free methods we used the case-control study on diabetes presented above in the section "Differential gene expression in human diabetes samples".

All the methods checked produced comparable results (see Table 1). *Oxidative phosphorylation *and *mitochondrion *are found by GSEA [16,17], PAGE [24] and Tian's [22] methods. *Nucleotide biosynthesis *can be assimilated to other, equivalent functional classes based on a set of functional categories developed by the authors of the GSEA [17], and both were found by all of the methods.

**Table 1:** Significant functional classes across a case-control study of diabetes.

		Repository				Method			
Healthy vs diabetic	Functional class	GO	KEGG	Swissprot keyword	Defined in GSEA	FatiScan	GSEA	PAGE	Tian et al.

Up-regulated	Oxidative phosphorylation	•	•		•	yes	yes	yes	yes
	ATP synthesis		•			yes	-	-	-
	Ribosome		•			yes	-	-	-
	Ubiquinone			•		yes	-	-	-
	Ribosomal protein			•		yes	-	-	-
	Ribonucleoprotein			•		yes	-	-	-
	Mitochondrion	•		•	•	yes	yes	yes	yes
	Transit peptide			•		yes	-	-	-
	Nucleotide biosynthesis	•			•	yes	yes	yes	yes
	NADH dehidrogenase (ubiquinone) activity	•				yes	-	-	-
	Nuclease activity	•				yes	-	-	-
Dow-regulated	Insulin signalling pathway		•			yes	-	-	-

Different functional classes, making reference to distinct functional terms, found as over-represented at the extremes of the list of genes ordered by differential expression between healthy controls versus diabetic cases (see text) by distinct threshold-free methods. Some of the functional classes refer to similar functional concepts and contain essentially the same genes, but have been defined in different repositories (GO, KEGG, etc.) FatiScan uses functional classes defined through GO, KEGG and Swissprot keywords (among other), while GSEA, PAGE and Tian et al. use the functional classes defined in GSEA [17].

The example showed how different methods efficiently detected similar functional classes that made reference to common functional properties of genes. And this detection was achieved even for the case of some of the functional classes that have been defined in different repositories and different contexts (and obviously with some differences in the composition of the genes). This agreement in the results is in bold contrast if compared to the poor overlap reported by different studies where the aim was the selection of genes (Bammler et al., 2005) instead of blocks of functionally related genes.

Nevertheless a more extensive and rigorous comparative study is still necessary to decide which is most efficient. Our aim was not to perform exhaustive benchmarking but just to show the basic agreement in the results obtained through different alternative threshold-free approaches.

From a practical point of view, the preferred methods would be those which can use more biological terms and software packages, such as FatiScan or GSEA [17]. Actually we have also implemented the GSEA in our Babelomics suite [29].

## Conclusion

Threshold-free approaches to the functional interpretation of genome-scale experiments are far more efficient than the "classical" threshold-based, two-step approaches. Following this philosophy, different tests that focus on blocks of functionally-related genes, instead on individual genes, have been proposed in the context of microarray data [16,17,19-24,65]. These tests study the over- or under-expression of blocks of functionally related genes by studying their relative position across a lists of genes ranked by differential expression. Nevertheless, such tests have hardly been implemented in user-friendly programs and have never been used outside of the context of gene expression.

The program presented here, FatiScan, implements a segmentation test [19] which does not require the original data to estimate the significance of the results found. The advantage of this test is that it is data-independent and consequently can be applied to any type of list generated in any genome-scale experiment of any nature.

Within the context of microarray data analysis FatiScan can be used to test different types of data given that the test is not based on assumptions based on the way in which the list is ordered. Thus either categorical variables (e.g. case-control experiments) of continuous variables (the level of a metabolite or survival) can be used to build up a list of genes ordered according their relationship to the variables studied. This property constitutes an advantage over some model-based approaches that need to distinguish between discrete [21] and continuous variables such as survival [20].

Actually, the combined use of biological information and experimental results is a possible solution to several recurrent problems in the field of microarray data analysis, such as the difficulties in extrapolating results across different platforms. A recent study in which the same experiment was performed in different laboratories using different platforms [15] has demonstrated that, in spite of the low concordance in the individual genes, the biological themes found were always the same. The application of approaches such as FatiScan or similar methods will overcome the classical problems derived from the two-step approaches.

In other fields, such as evolution, we have shown how the application of the FatiScan program circumvents the problems of lack of significance encountered by other authors. The test implemented in Fatiscan reported the functional classes and pathways suggested (but not statistically supported) by previous analyses as significant. The possibility of its application to fields other than microarrays is a completely original feature from FatiScan.

Summarising, FatiScan provides a convenient web-based environment for the functional analysis and interpretation of genome-scale experiments. FatiScan is part of the Babelomics suite [29,30] where many other related tools can be found. Moreover, the Babelomics is integrated in the GEPAS [28,34-36] suite for microarray data analysis, so if the genome-scale experiment is being made in the context of microarrays, many other tools including normalisation, data pre-processing, clustering, gene selection, predictors, array-CGH support, etc. are immediately available.

## Availability and requirements

Project name: FatiScan

Project home page:  and within 

Operating system: Platform independent web server

Programming language: Perl and R

## Abbreviations

**DAG: **Directed acyclic graph

**FDR**: False Discovery Rate

**GO**: Gene Ontology

**KEGG**: Kioto Encyclopaedia of Genes and Genomes

**NIA**: Nested Inclusive Analysis

## Authors' contributions

FAS is the author of the algorithm and the program and has participated in all the analyses, LA and HD have performed the evolutionary study, JHC has performed the study of the interactome, PM is co-author of the program, DM has carried out the study of survival and has supervised the statistical aspects of the manuscript and JD has conceived and coordinated the study and written the manuscript. All the authors have read and approved the final manuscript.
